# Mouse Models of Osteosarcoma: Unraveling Disease Mechanisms and Accelerating Drug Discovery and Development

**DOI:** 10.1002/cnr2.70390

**Published:** 2025-10-31

**Authors:** Staci L. Haney, Sarah A. Holstein

**Affiliations:** ^1^ Department of Internal Medicine University of Nebraska Medical Center Omaha Nebraska USA

## Abstract

**Background:**

Osteosarcoma is the most frequent primary bone malignancy, affecting mainly children, adolescents, and young adults. For the past 40 years, improvements in the survival of patients with osteosarcoma have been minimal, primarily as a consequence of the lack of systemic therapy options beyond traditional cytotoxic agents. In particular, management of metastatic and recurrent disease continues to be a significant clinical challenge.

**Recent Findings:**

Mouse models of osteosarcoma serve as a valuable tool to study disease biology, metastasis, and response to novel treatments. In recent years, mouse models have been employed to evaluate the efficacy of several innovative drugs, including nanoparticle formulations that can target drug delivery to osteosarcoma tumor cells and diminish off‐target effects. In addition, significant preclinical advancements in immune checkpoint inhibitors and immunotherapies for osteosarcoma have been made with the aid of mouse models.

**Conclusion:**

This review provides insight into the advantages and shortcomings of the numerous osteosarcoma mouse models described in the literature, including transgenic, orthotopic, and heterotopic models as well as patient‐derived xenografts. We highlight how these models are currently being used to support preclinical studies focused on the development of novel therapies.

## 1 Introduction

Osteosarcoma (OS) is the most common form of bone cancer and occurs most frequently in children and young adults aged 10–24 years old, with a secondary smaller peak in adults after age 65 [1]. Overall, OS is a relatively rare malignancy, accounting for 3% of childhood cancers [2]. Males are more frequently diagnosed with osteosarcoma and experience worse overall survival across all age brackets relative to females [3]. OS typically develops in the metaphysis of long bones near the growth plate, with the distal femur and proximal tibia being the most affected locations. Frontline treatment for OS includes intensive neoadjuvant chemotherapy and surgical resection of the tumor. The 5‐year survival rate for patients with localized disease is approximately 70%, while survival for those with distant metastasis drops to less than 30% [4, 5, 6]. Approximately 15%–20% of patients will have detectable metastatic disease at the time of diagnosis, with 85% of metastatic cases being in the lung [7, 8]. While many patients show a favorable initial response to treatment, relapse rates are high at nearly 25% and prognosis is poor for those who experience recurrence [9]. Unfortunately, despite intensive preclinical efforts and numerous clinical trials, survival rates for OS have remained largely unchanged for the past four decades [10]. Novel treatment options, such as immunotherapies and targeted agents, that have proven beneficial in treating other cancer types have not been successfully implemented for OS. Thus, there is an urgent need for the development of novel therapeutic strategies for the treatment of OS. Preclinical studies with reliable mouse models that closely recapitulate human disease are critical in achieving this goal.

**FIGURE 1:**
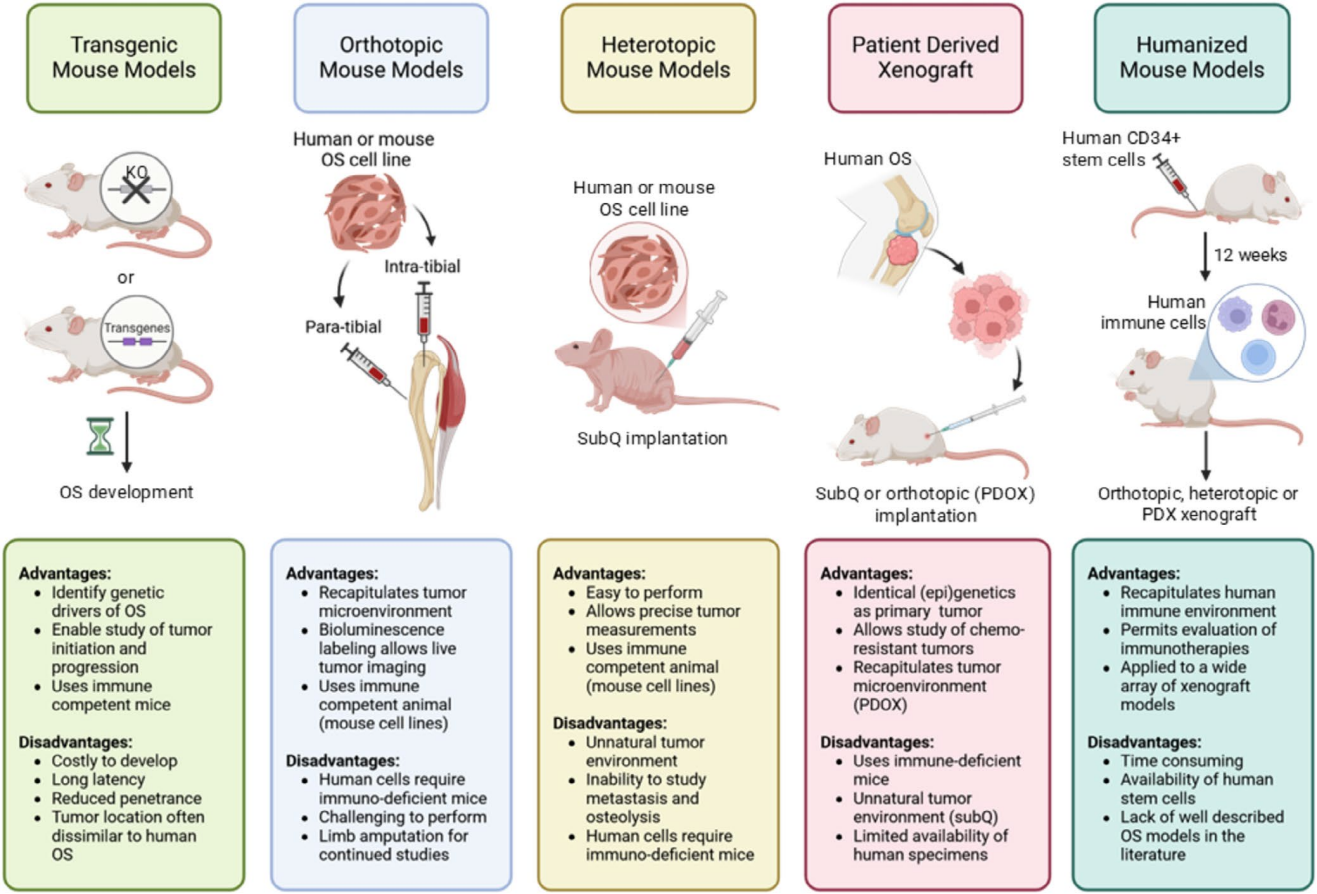
Overview of osteosarcoma mouse models. Advantages and disadvantages of the five categories of OS mouse models are shown, along with a graphical depiction of each model. PD(O)X = patient derived (orthotopic) xenograft, SubQ = subcutaneous. This figure was created using BioRender.

Spontaneous development of OS occurs in < 1% of mice, thus mouse tumor models are essential for the study of disease progression, metastasis, and drug response [11]. An ideal preclinical model should not only mimic human OS genetic and histological features but also allow for timely evaluation of drug response and toxicity. Ultimately, finding a model with translational potential should be the goal. Here we present a narrative review of mouse models of OS that are described in the literature, including transgenic, orthotopic, and heterotopic models, as well as patient‐derived xenografts (PDX), and draw conclusions about each model's advantages and shortcomings as they pertain to drug discovery (Figure 1). This review also highlights how these models are currently being used in preclinical studies to develop novel therapies. The search strategy for this review utilized PubMed and the following search terms: osteosarcoma plus mouse models, transgenic mice, xenograft, preclinical, para‐tibial, intra‐tibial, flank xenograft, PDX, PDOX, humanized mice, nanoparticles, and immunotherapy. Recent advances in preclinical drug development were limited to articles published within the last 5 years, while articles pertaining to mouse model development had no time filters applied.

## 2 Transgenic Mouse Models

Transgenic mouse models (TMMs) have several benefits, including the ability to manipulate the expression of specific genes to identify drivers of human disease. They also allow study of oncogenesis, disease progression, and drug response in an immune‐competent animal. Despite their strengths some TMMs also have disadvantages, such as long latency periods, reduced penetrance, and high cost to develop. Furthermore, dissimilarities with human OS, including metastatic potential and location of tumor development limit their use in preclinical studies.

In 1993, the first TMM for OS, the H2‐fosLTR mouse, was developed [12]. This model relies on overexpression of the proto‐oncogene *c‐fos* to promote tumorigenesis. The *c‐fos* transgene is fused to the HK‐2 class II MHC promoter, and despite broad tissue expression of *c‐fos*, mice exclusively develop OS with osteoblasts identified as the cell of origin (Table 1). OS development occurs rapidly, with tumors present on radiographs as early as 4 weeks of age. Unfortunately, this model lacks metastatic potential, likely due to the rapid disease progression and short lifespan of the mice, which limits its usefulness in drug development.

**TABLE 1:** Transgenic Models for OS.

Genotype	Penetrance	Latency	Tumor location	Lung metastasis	Cit.
*H2k‐fos‐Tg*	100%	4 weeks	Limb bones	No	[12]
*Prx1‐cre;P53* ^*lox/lox*^	61% (OS) 32% (PDSTS)	1 year	Limb bones (74%), pelvis (11%), spine, ribs or scapula (15%)	Yes	[13]
*Prx1‐cre;P53* ^*lox/+*^	22% (OS) 17% (PDSTS)	96 months	Limb bones	Not noted	[13]
*Prx1‐cre;p53* ^*lox/lox*^ *;Rb* ^*lox/lox*^	29% (OS) 57% (PDSTS)	19 weeks	Not noted	Yes	[13]
*Col1a1‐cre;p53* ^*lox/lox*^	85% (OS) 15% (PDSTS)	10.5 months	Not noted	Not noted	[13]
*Col3.6‐Cre, p53* ^*lox/lox*^	60% (OS) 20% (lymphoma) 20% (fibrosarcoma)	9 months	Not noted	Not noted	[14]
*Osx1‐Cre;Rb1* ^*+/lox*^ *;p53* ^*lox/lox*^	94%	7 months	Cephalic (primary), femur (secondary)	Yes	[15, 16]
*Osx1‐Cre;Rb1* ^*lox/lox*^ *;p53* ^*lox/lox*^	100%	4–5 months	Cephalic (78%), limb bones (14%), rib/sternum (8%)	Yes	[15, 16]
*Osx1Cre* ^+^ *;Wwox* ^*fl/+*^ *p53* ^*fl/fl*^	94%	4 months	vertebrae (30%), limbs and hip (45%), jaw (25%)	Yes	[17]
*Cre‐Lox‐Stop‐Lox‐c‐Myc* ^*T58A*^ *;Trp53* ^*fl/+*^	100% (OS)	6 months	Not noted	Yes	[18]
*Col1a1 2.3 kb‐Cre; Rosa26* ^*NICD*^ (NICD)	36% (OS) 18% (Osteoma) 46% (OS+Osteoma)	10 months	Limb bones (69%), rib (13%), hip (10%), spine (7%), skull (2%)	Yes	[19]
*Col1a1 2.3 kb‐Cre;Rosa26* ^*NICD*^ *; Trp53* ^*Flox/Flox*^	83%	5 months	Limb bones (37%), spine (27%), hip (17%), rib (13%), skull (6%)	Yes	[19]
Apc1638^N/+^;twist^null/+^	33%	15 months	Cephalic	No	[20]
*Ptch1* ^*c/+*^ *;p53* ^*+/−*^ *;HOC‐Cre*	70%	7–12 months	Limb bones	Yes	[21]

Abbreviation: PDSTS, poorly differentiated soft tissue sarcoma.

The majority of the TMMs for OS rely on heterozygous or homozygous conditional deletion of the *Trp53* gene, which encodes the tumor suppressor protein p53 (Table 1). Early studies suggested *Trp53* was mutated in about 22% of all human OS cases, while subsequent whole genome sequencing revealed that inactivating mutations within the p53 pathway were present in 95% of all OS tumors [22, 23]. The prevalence of *Trp53* inactivation correlates with the high degree of genomic instability observed in OS [24]. Homozygous conditional deletion of *Trp53* in either mesenchymal stem cells (*Prx1‐cre;P53* ^*lox/lox*^) or osteoblasts (*Col1a1‐cre;p53* ^*lox/lox*^ *or Col3.6‐Cre;p53* ^*lox/lox*^) promotes OS development in mice with a latency period of approximately 9 months to 1 year [13, 14]. Heterozygous deletion of *Trp53* (*Prx1‐cre p53* ^*lox/+*^) also leads to OS development in mice but lengthens latency to approximately 2 years. Thymic lymphoma, lung carcinoma, squamous cell carcinoma and mammary carcinoma were also observed after heterozygous *Trp53* deletion [13]. OS tumors primarily arose in the limb bones, which is an advantage of this model as it mimics human OS development [13]. While these models provide insight into the molecular pathways that underlie OS initiation and progression, the long latency period limits their utility in drug development research.

Additional strategies for TMM development involve combining p53 deficiency with loss of another tumor suppressor gene, such as *Wwox* or *Rb1*, to potentiate OS development and shorten latency (Table 1). Conditional deletion of *Wwox and Trp53 (Osx1Cre* ^+^ *;Wwox* ^*fl/+*^ *p53* ^*fl/fl*^ *) in osteoblasts* leads to the development of OS in mice within 4 months [17]. Likewise, conditional double knockout (DKO) of *Trp53* and *Rb1* in either mesenchymal stem cells (*Prx1‐cre p53* ^*lox/lox*^ *Rb* ^*lox/lox*^) or osteoblasts (*Osx1‐Cre;Rb1* ^*lox/lox*^ *;p53* ^*lox/lox*^) leads to OS development in mice within 4–5 months [13, 15, 16]. The *Osx1‐Cre;Rb1* ^*lox/lox*^ *;p53* ^*lox/lox*^ TMM recapitulates many defining features of human OS, such as cytogenetic complexity, transcriptional profiles, and histological features [15]. Interestingly, while tumor development is observed in the limb bones of some mice, the majority of *Trp53/Rb1* DKO mice (76%) develop tumors in locations outside of those typically observed in human OS, such as the jaw, snout and head [15]. The *Trp53/Rb1* DKO mice also display a lower incidence of metastasis compared to the single *Trp53* knockout [15]. Furthermore, 50% of mice with homozygous deletion of both *Rb1* and *Trp53* in osteoblasts developed tumors of a neuroendocrine origin, suggesting that the *Osx1‐Cre* transgene is expressed at low levels in neuroendocrine tissues or precursors [16]. Heterozygous deletion of *Rb1* (*Osx1‐Cre;Rb1* ^*+/lox*^ *;p53* ^*lox/lox*^) lengthens latency but reduces the incidence of neuroendocrine tumors. OS was not observed after loss of *Rb1* alone, indicating that the *Trp53* mutation is critical, while the *Rb1* mutation only potentiates OS development.

The MYC oncogene, also known as c‐MYC, belongs to a superfamily of transcriptional regulators that control the expression of numerous protein‐coding and noncoding genes involved in essential cellular processes such as cell cycle regulation, signal transduction, metabolism, transcription, and protein synthesis [25]. Aberrations in the *MYC* locus or deregulation in *MYC* pathways are identified in more than half of all human cancers [26]. Approximately 20% of OS tumors contain a duplication of the 8q24 locus, which harbors the *c‐MYC* oncogene [27, 28]. To elucidate the role of *c‐MYC* in OS development, Nirala et al. generated an osteoblast‐specific c‐*MYC* knock‐in TMM (*Cre‐Lox‐Stop‐Lox‐c‐Myc* ^*T58A*^ *;Trp53* ^*fl/+*^, Table 1) [18]. The *c‐MYC* knock‐in mice rapidly developed OS (median survival of 24 weeks) with a high incidence of pulmonary metastasis (> 60%). Furthermore, overexpression of c‐*MYC* led to increased microRNA 17/20a expression which caused downregulation of macrophage colony‐stimulating factor 1 and reduction in tumor infiltrating macrophages. These results provide mechanistic insight into how *MYC* signaling permits tumor cells to disrupt their microenvironment and escape detection by the immune system.

Several p53‐independent TMMs have been developed (Table 1). These models exploit oncogenic drivers, such as Notch1, APC, and hedgehog signaling. The *Col1a1 2.3 kb‐Cre; Rosa26* ^*NIC*^ (NICD) transgene drives the overexpression of the intracellular domain of Notch1 in osteoblasts [19]. The NICD mice were smaller in size than their wild‐type littermate and often died of tumor‐unrelated causes by 3 months of age. However, 100% of the surviving NICD mice spontaneously developed either OS, osteoma or both between the ages of 5 and 14 months. Tumor initiation and progression in NICD mice are accelerated by deletion of *Trp53* (*Col1a1 2.3 kb‐Cre*; *Rosa26* ^*NICD*^; *Trp53* ^*Flox/Flox*^) and these tumors shared important characteristics with human OS, including histopathology, cytogenetic complexity, and metastatic potential. Examination of a cohort of pediatric OS patients revealed frequent and concurrent loss of *TWIST* and *APC* genes, which are important regulators of bone development [29]. Mice with both haploinsufficiency for *TWIST* and heterozygous expression of mutated *Apc* ^*1638N*^ allele (*Apc1638* ^*N/+*^; *twist* ^*null/+*^) developed OS tumors with incomplete penetrance and a relatively long latency (> 15 months in many cases) [20]. Most mice developed tumors in the cephalic regions. Given the relevance of hedgehog signaling in tumor development, Chan et al. generated a mouse model with upregulated hedgehog signaling in mature osteoblasts and crossed it into a *Trp53* heterozygous background to potentiate tumor development [21]. The resulting mice (*HOC‐Cre*; *Ptch1* ^*c/+*^; *p53* ^*+/−*^) developed visible tumors in the forelimbs, tibia, and femur, with little involvement in the skull. While these models provide insight into the molecular drivers of OS, they have not been utilized in drug development efforts, likely due to their long latency and reduced penetrance.

## 3 Orthotopic Mouse Models

Orthotopic mouse models are accomplished by the injection of OS cells either adjacent to the bone (para‐osseous) or directly into the femur or tibial diaphysis (intra‐osseous). The intra‐osseous model best recapitulates the tumor microenvironment observed in human OS but is technically the most challenging to accomplish and requires anesthesia. These models are generated with the injection of mouse OS cell lines into syngeneic mice (example: K7M2 cells in BALB/C mice) or human OS cell lines into immunocompromised mice (example: 143B cells in Nude or NOD/SCID mice). OS cell lines are often engineered to express luciferase to allow bioluminescent imaging and monitoring of tumor growth. The abundant variety of OS cell lines available for study and the detailed characterization of orthotopic models in the literature are key advantages.

Several cell lines have been established from OS tumors that arose spontaneously in wild‐type mice (Table 2). Establishment of orthotopic mouse models with these cell lines allows researchers to study OS in an immunocompetent animal, which is critical for the evaluation of novel immune‐modulating drugs. K7M2 cells form rapid tumors when injected into the tibia of C57Bl/6 mice and show high metastatic potential to the lungs [30, 31]. Importantly, early surgical removal of the inoculated limb did not impact metastatic burden [30]. An additional model involves the injection of MOS‐J cells in syngeneic C57Bl/6 mice [32]. Crenn et al. compared the therapeutic response of three commonly used chemotherapy agents (doxorubicin, cisplatin, ifosfamide) in both intra‐ and para‐tibial established MOS‐J tumors [33]. A significant benefit in therapeutic response to doxorubicin was observed in mice with intra‐osseous tumors relative to para‐osseous tumors, indicating that the microenvironment plays a role in how OS responds to chemotherapy. These results highlight the importance of using intra‐osseous models in drug development when possible.

**TABLE 2:** Orthotopic and heterotopic models using mouse OS cell lines.

Cell line	Route of injection	Mouse strain	Metastasis	Osteolytic	Citation
K7M2	Intra‐tibial	BALB/C	Yes	Not noted	[30, 31]
MOS‐J	Para‐ and intra‐tibial	C57Bl/6	Yes	Yes	[32, 33, 34]
LM8	SubQ Intra‐tibial	CH3/HeN	Yes	Not noted	[35, 36]
DLM8‐M1	Intra‐tibial	CH3/HeN	Yes	Not noted	[37]
POS‐1	Para‐tibial SubQ	CH3/HeN	Yes	Yes	[38, 39]

Abbreviation: SubQ, subcutaneous.

The Dunn cell line was generated from a spontaneous OS tumor that developed in a CH3/HeN mouse and has been utilized to develop several additional cell lines (LM8, DLM8‐M1, and POS‐1) [35, 40, 41]. LM8 cells were selected for their high metastatic seeding to the lung, which occurs even after subcutaneous (subQ) transplantation [35]. DLM8‐M1 cells also have high metastatic potential and form tumors within 5 weeks when injected intra‐tibial in CH3/HeN mice [37]. POS‐1 cells have been used in both subQ and para‐osseous xenograft [38, 39].

Orthotopic models using human OS cell lines are routinely utilized in drug discovery research (Table 3). The HOS cell line was established in 1971 from the tumor of a 13‐year‐old patient with OS and over the years has been modified in vitro giving rise to several cell line variants, including 143B, KRIB, MNNG/HOS, and KHOS [61]. The 143B and KRIB cells are both highly metastatic OS cell lines that when injected intra‐osseous will disseminate to the lung, thus allowing researchers to study the therapeutic effects of novel agents on metastatic disease [42, 51]. The primary disadvantage of these models is their aggressiveness, giving researchers a narrow window with which to study drug response. For instance, KRIB cells injected intra‐tibial produce palpable tumors within 2 weeks and lung metastases by 6 weeks [51]. To lengthen survival time the primary tumor must be removed surgically by 8 weeks. Similarly, mice injected intra‐tibial with 143B cells reach a terminal end point by 4 weeks [37]. Both MNNG/HOS and KHOS cell lines readily form tumors in Nude mice when injected para‐tibial [39, 49]. Importantly, MNNG/HOS, 143B and KHOS cells form highly osteolytic lesions, allowing researchers to evaluate a therapy's impact on OS‐mediated bone loss. Additional human OS cell lines have also been utilized in orthotopic mouse models, including MG63, SaOS‐2, LM7 and G‐292 [37, 43, 47, 52, 60, 62].

**TABLE 3:** Orthotopic and heterotopic models using human OS cell lines.

Cell line	Route	Mouse strain	Metastasis	Osteolytic	Citation
143B	Intra‐tibial	Nude, NOD‐SCID	Yes	Yes	[37, 42, 43, 44]
	SubQ	Nude, NSG	No	No	[45, 46]
Cal72	SubQ	NCG	No	No	[45]
G‐292	Intra‐tibial	SCID	Yes	Yes	[47]
	SubQ	NCG	No	No	[45]
HAL	SubQ	NCG	No	No	[45]
HOS	SubQ	NCG, Nude	No	No	[45, 48]
KHOS	Intra‐tibial	Nude	Yes	Yes	[49, 50]
KPD	SubQ	NCG	No	No	[45]
KRIB	Intra‐tibial	Nude	Yes	Yes	[50, 51]
LM7	Intra‐tibial	NOD‐SCID	Yes	No	[43, 52]
MG63	Intra‐tibial	Nude, NOD‐SCID	Rare	No	[43, 53]
	SubQ	Nude, NSG	No	No	[45, 54]
MHM	SubQ	NCG	No	No	[45]
MNNG/HOS	Para‐tibial	Nude	Yes	Yes	[55]
	Intra‐tibial	NSG	Yes	Yes	[56]
	SubQ	Nude, NSG	No	No	[45, 46]
OHS	SubQ	NCG	No	No	[45]
OSA	SubQ	Nude, NSG	No	No	[45, 46]
SJSA‐1	SubQ	Nude	No	No	[57, 58, 59]
SaOS‐2	Intra‐tibial	NOD‐SCID, NSG	Rare	Not noted	[37, 43, 60]
	SubQ	Nude	No	No	[45]
ZK‐58	SubQ	NCG	No	No	[45]

Abbreviations: NCG, *NOD‐Prkdc* ^*em26Cd52*^ *Il2rg* ^*em26Cd22*^ */NjuCrl*; OD‐SCID, NOD.Cg‐*Prkdc* ^*scid*^/J; NSG, *NOD.Cg‐Prkdcscid Il2rgtm1Wjl/SzJ*; Nude—athymic nude mice (Crl:NU(NCr)‐*Foxn1* ^*nu*^), CD‐1 nude mice (Crl:CD1‐*Foxn1* ^*nu*^), or BALB/c nude mice (CAnN.Cg‐*Foxn1* ^*nu*^/Crl); SubQ = subcutaneous.

## 4 Heterotopic Mouse Models

Heterotopic models involve the injection of tumor cell lines into the subQ (most commonly the flank) space of immunocompromised mice (Table 3). While these models do not recapitulate the tumors' natural environment, they allow more precise measurement of tumor growth and are relatively easy to perform and replicate. In general, heterotopic models typically do not exhibit metastatic potential. One notable example, the SJSA‐1 cell line, readily grows in the flank of Nude mice and is a useful tool for studying drug efficacy [57, 58, 59]. Lauvrak et al. evaluated the in vivo growth of 22 human OS cell lines by inoculating NCG mice with 1 million OS cells in the flank [45]. The cell lines were placed in groups based on their growth rate in mice, including highly tumorigenic cell lines (OSA, 143B, HOS, HOS‐MNNG, MHM, OHS), tumorigenic (Cal72, HAL, ZK‐58, KPD, G‐292, SaOS‐2), lowly tumorigenic (MG63, IOR/OS15, U2OS) non‐tumorigenic cell lines (IOR/OS18, IOR/OS10, IOR/SARG, 11 T245, IOR/MOS).

## 5 PDX Models

Patient‐derived xenografts (PDX) involve the transplantation of human primary OS tumors into immunocompromised mice. This model is extensively utilized in the investigation of OS therapeutics, with over 100 compounds evaluated via PDX models in recent years [63]. Typically, these models are accomplished by either implantation of a small piece of tumor tissue (2 mm^3^) subQ into the flank or by injection of enzymatically separated tumor cells into para‐ or intra‐osseous sites. The primary advantage of PDX models is that they carry identical genetic and epigenetic modifications as the primary human tumor. They also allow evaluation of novel agent efficacy in tumors with known resistance to standard therapeutics. Limitations of these models include a lack of availability, poor engraftment, and an inability to replicate the immunological complexities of human OS.

Importantly, the efficacy observed within these models may not reliably predict a therapeutic benefit in clinical trials. This was the case for the antibody‐drug conjugate glembatumumab vedotin and chemotherapy agent eribulin, both of which showed a reduction in tumor growth in PDX models but failed to show a significant response in human clinical trials [64, 65]. Similarly, trastuzumab deruxtecan (T‐Dxd), a humanized monoclonal HER2‐targeting antibody conjugated to a topoisomerase 1 inhibitor, was shown to slow tumor growth in 6/7 PDX [66]. However, a phase II clinical trial evaluating the effectiveness of T‐Dxd in patients with HER2+ OS (NCT04616560) failed to demonstrate a sufficient response; thus the study was terminated [67]. Lack of agreement between preclinical data and clinical trial outcomes could be due to a variety of factors, such as interactions with the immune system, heterogeneity of the tumor, and differences in pharmacokinetics, biodistribution, or toxicity profiles between mice and humans.

Heterotopic implantation of PDX xenografts may also be to blame for the false positives (or negatives) observed in preclinical studies, as this method fails to recapitulate the tumors' original microenvironment. Engraftment of PDX tumors into or around the bone (often referred to as PDOX models) has been successfully employed in various pre‐clinical drug development studies for OS [68, 69, 70, 71, 72]. In contrast to subQ flank tumors, implantation of intact human tumor samples into the tibia allows detection of distant metastasis in the majority of mice [73]. Overall, PDX models are a valuable tool for researchers, but they should not be exclusively relied on for the prediction of clinical success.

**FIGURE 2:**
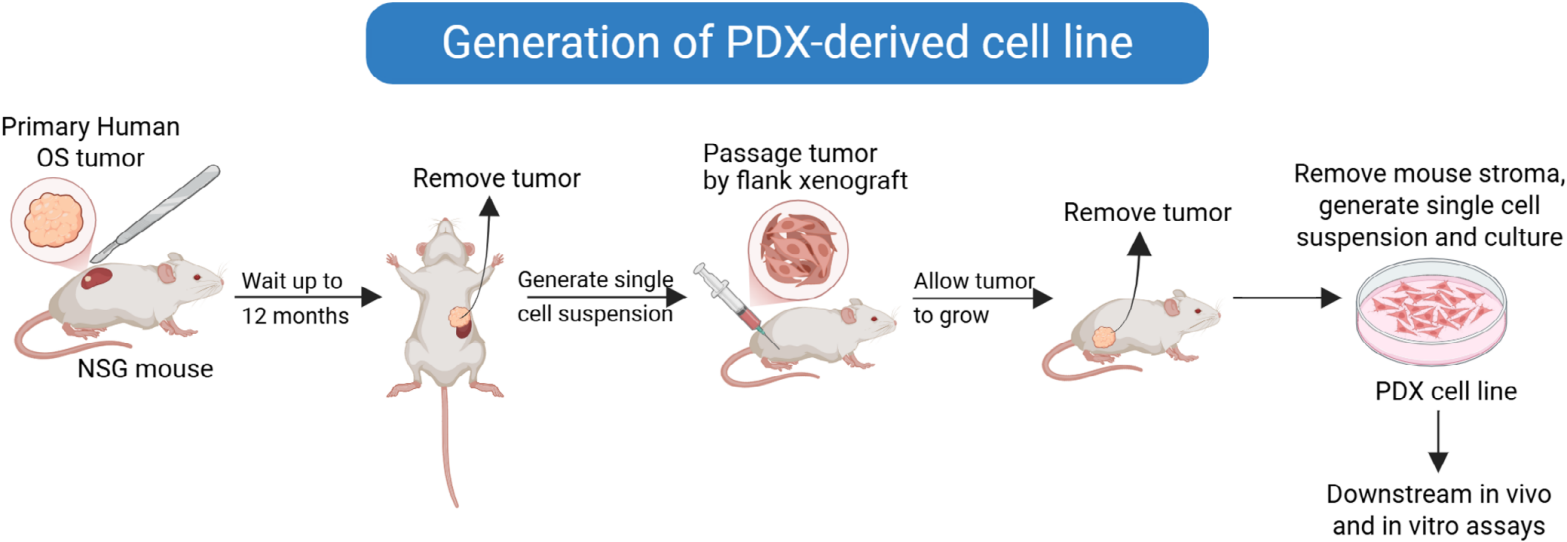
Generation of PDX‐derived cell lines. Schematic showing how PDX‐derived cell lines are produced, beginning with a primary human OS tumor sample implanted into the renal capsule of an NSG mouse. After sufficient growth, tumors are removed and passaged via flank xenograft, before being isolated again and grown in culture. This figure was created using BioRender.

Concerns regarding the ability of highly passaged OS cell lines to reliably reproduce human OS characteristics prompted the development of PDX‐derived tumor cell lines [74]. In these studies, human OS samples were first implanted into the renal capsule of NOD scid gamma (NSG) mice and after sufficient growth tumors were isolated and passaged via flank xenograft before being grown in culture (Figure 2). Importantly, the resulting PDX cell lines exhibited similar gene expression patterns and genomic landscapes when compared to primary tumors. The PDX‐derived cell lines were heterogeneous in their metastatic capacity in an orthotopic amputation model. Furthermore, dinaciclib, a CDK2/5/9 inhibitor, was effective at reducing the metastatic burden in mice transplanted with a PDX‐derived cell line that carried a MYC amplification. While these cell lines recapitulate important characteristics observed in OS patient samples, including genetic landscapes and metastatic ability, the high cost and extensive time required to develop them limit their accessibility within the research community.

## 6 Humanized Mouse Models

Due to differences in human and mouse immune systems, even immunocompetent mouse models may not accurately recapitulate the immune environment that influences human OS development and response to treatment. This fact has led researchers to employ humanized mouse models, whereby immunocompromised mice are reconstituted with human immune cells before tumor xenografts are established. Ko et al. successfully developed a humanized mouse model for OS using NSG mice engrafted with human CD34+ hematopoietic stem cells [75]. They show that KHOS/NP tumor growth is influenced by higher levels of human immune cell engraftment, specifically T cells. In another study using humanized mice and KHOS heterotopic xenografts, researchers showed increased tumor infiltration of CD4+ and CD8+ T cells and reduced lung metastasis in mice treated with the immune checkpoint inhibitor nivolumab, although no effect on primary tumor growth was observed [76]. Comparable humanized mouse models employing CD34^+^ stem cells and tumor cell lines are used to investigate other bone and soft tissue sarcomas, including Ewing sarcoma [77, 78].

To develop a clinically relevant human bone microenvironment, Wagner et al. generated humanized tissue‐engineered bone organs (hTEBO) by implanting human pelvic bone and marrow fragments embedded with bone morphogenetic protein‐7 (BMP‐7) fibrin matrix into NOD‐SCID mice [79]. Flow cytometry confirmed that human hematopoietic stem cells and leukocytes survive within the humanized microenvironment. After 10 weeks of subQ growth, hTEBOs were injected with human luciferase‐labeled SaOS‐2 cells. Lung metastasis was observed and prognostic markers of the tumors, including VEGF, periostin and HIF2α expression, were consistent with human OS. Overall, this model reduces interspecies differences between humans and mice and recapitulates the OS bone microenvironment, offering a novel model for translational research.

To the best of our knowledge, humanized PDX/PDOX mouse models for OS have not been described in the literature but represent an important next step in the development of reliable mouse models. While the utilization of humanized mice in OS preclinical studies is still in its infancy, they represent a powerful tool to study the interactions between human immune cells and tumors and permit evaluation of novel immunotherapies.

## 7 Recent Advances in OS Preclinical Drug Discovery

While most patients show favorable initial responses to traditional cytotoxic chemotherapy, treatment often fails due to the development of drug resistance and unwanted side effects. There is considerable interest in the field of drug development in utilizing nanoparticle (NP) formulations that can target drug delivery to tumor cells and diminish off‐target effects. This strategy was employed to create a novel folate receptor‐targeted doxorubicin (DOX) and edelfosine‐loaded lipid–polymer hybrid [80]. The targeted NPs showed enhanced OS cell internalization and significantly suppressed tumor growth in an MG‐63 flank xenograft relative to non‐targeted NPs. In a similar study, DOX‐loaded nanoparticles labeled with a vimentin binding peptide to guide OS cell targeting were evaluated in vivo [81]. These NP formulations significantly decreased tumor burden in 143B xenografts relative to controls and reduced the toxicities of DOX to major organs. In another study, a therapeutic gadolinium‐based metal‐bisphosphonate NP (OVA‐GdZol NPs) was generated and combined with radiotherapy in the K7M2 orthotopic mouse model [82]. OVA‐GdZol NPs and X‐ray radiation reduced tumor volume, lengthened survival, and decreased lung metastasis in mice, while also promoting the maturation of bone marrow‐derived dendritic cells and M1 polarization of macrophages. By serving as a radiosensitizer, this approach has the potential to overcome the radiation resistance observed in OS. Lastly, bone‐targeted nanoparticles carrying NF‐κB essential modulator binding domain peptides were recently developed and evaluated in vivo for osteosarcoma treatment [83]. The targeted NPs efficiently inhibited tumor growth and osteosarcoma‐induced bone destruction in 143B orthotopic xenografts. Taken together, NPs are a promising approach for the development of targeted therapy.

The development of immune checkpoint inhibitors and immunotherapies for OS is greatly hindered by the malignancy's low immunogenicity and immunosuppressive microenvironment. In a novel approach, Ge et al. developed a NP formulation termed CBZP that contained curcumin for autophagy activation and BMS1166 for inhibition of the PD‐1/PD‐L1 axis [84]. Not only did CBZP drastically reduce tumor growth in a K7M2 orthotopic mouse model, but prior CBZP treatment conferred strong immune memory effects in mice re‐challenged with K7M2 cells. It is noted that prior efforts to employ PDL‐1 inhibitors for OS treatment failed across several clinical trials [85]. Perhaps combining induction of immunogenic cell death (via autophagy induction) with immune checkpoint blockade (via PDL‐1 inhibitors) can provide the synergy needed to effectively employ PDL‐1 therapy to treat OS. While the above examples highlight recent advances in NP drug development for OS, there are several additional studies suggesting NPs have superior anticancer activity across a range of mouse models of OS [83, 86, 87, 88, 89].

There is considerable interest in developing therapies to target tumor‐associated macrophages (TAMs), due to their tumor‐promoting characteristics, such as their ability to secrete proangiogenic growth factors and immunosuppressive cytokines [90]. Colony‐stimulating factor 1 (CSF‐1) is a primary regulator of the survival, proliferation, and differentiation of TAMs. In preclinical studies, PLX33987, a CSF‐1 inhibitor, was shown to suppress primary tumor growth and lung metastasis in both orthotopic (LM8 and OSA) and PDX mouse models [36, 91]. Furthermore, PLX3397 treatment depleted both TAMs and FOXP3^+^ regulatory T cells, as well as increased infiltration of CD8^+^ T cells at both primary and metastatic OS sites [36]. PLX3398, also known as pexidartinib, was recently approved by the FDA for treatment of tenosynovial giant cell tumor (TGCT) [92]. While there are currently no clinical trials underway for pexidartinib in OS, the preclinical data suggest the drug has potential as a novel therapy for OS.

Several small molecule inhibitors have been developed in recent years and evaluated in OS mouse models. Chessari et al. identified two potent, selective, and orally efficacious inhibitors of the p53‐MDM2 protein–protein interaction [57]. Both inhibitors displayed favorable pharmacodynamic effects and antitumor efficacy in the SJSA‐1 heterotopic xenograft model. In another study, TH1579, a small molecule inhibitor of MTH1, slowed tumor growth and reduced the development of pulmonary metastases in a MNNG/HOS para‐tibial xenograft model [55]. Lastly, Zou et al. identified 4‐acetylcytidine acetyltransferase 10 (NAT10) as a candidate therapeutic target, which they note correlated with a poor prognosis in patients with OS [93]. They demonstrated that inhibition of NAT10 suppresses OS progression in PDX models. Collectively, these studies identify novel targets for pharmacological inhibition that may serve as feasible therapeutic approaches for OS.

## 8 Conclusion

The primary objective of OS preclinical research is to develop therapies that can be translated to the clinic and benefit patients. Mouse models are an indispensable tool for studying OS development, progression, metastasis, and response to therapy. While a variety of mouse models have been described in the literature, not all are uniquely suited to drug development research. Factors like latency, penetrance and tumor location limit the usefulness of transgenic mouse models in the field of drug discovery. Orthotopic and heterotopic models using mouse or human OS cell lines, as well as PDX models are a mainstay of OS preclinical research. However, they have their limitations, as they often fail to recapitulate the intricate tumor and immune microenvironments observed in human disease.

Overall, using a single mouse model is not the best approach for studying malignancy characterized by complex genetic landscapes and phenotypes. Furthermore, evaluation of a drug in a single mouse model is unlikely to predict patient outcomes in downstream clinical trials. While such false positives have been documented, this limited approach is likely to generate false negatives as well. Regarding pre‐clinical drug development, complementary use of multiple mouse models is encouraged, with emphasis on models that provide either a bone microenvironment (orthotopic, TMM, PDOX), immunocompetent animals (syngeneic orthotopic or heterotopic, TMM) or ideally both (humanized orthotopic models). PDOX models should be prioritized over PDX models, when possible.

Moving forward, continued development of mouse models that accurately replicate the unique tumor microenvironment and immune interaction of human OS is critical for translational research. Further advancement of humanized mouse models will help decipher the role of human immune cells in OS tumorigenesis and response to therapy. Humanized mice may also permit continued development of immunotherapies, which have proven immensely successful in the treatment of other cancers but are rather underexplored in OS. Lastly, future research harnessing emerging technologies, such as CRISPR/Cas9 gene editing and single‐cell sequencing, could allow for the identification of targets that may be pharmacologically exploited to develop novel therapies.

## Author Contributions

**Staci L. Haney:** conceptualization (lead), investigation (lead), writing – original draft (lead). **Sarah A. Holstein:** conceptualization (supporting), supervision (lead), writing – review and editing (lead).

## Ethics Statement

The authors have nothing to report.

## Conflicts of Interest

The authors declare no conflicts of interest.

## Data Availability

Data sharing is not applicable to this article as no new data were created or analyzed in this study.
